# Sensitivity of direct versus concentrated sputum smear microscopy in HIV-infected patients suspected of having pulmonary tuberculosis

**DOI:** 10.1186/1471-2334-9-53

**Published:** 2009-05-06

**Authors:** Adithya Cattamanchi, David W Dowdy, J Lucian Davis, William Worodria, Samuel Yoo, Moses Joloba, John Matovu, Philip C Hopewell, Laurence Huang

**Affiliations:** 1 Division of Pulmonary and Critical Care Medicine, University of California, San Francisco, CA, USA; 2 MU-UCSF Research Collaboration, Kampala, Uganda; 3 MU-UCSF Research Collaboration, CA, San Francisco, USA; 4 Francis J. Curry National Tuberculosis Center, San Francisco, CA, USA; 5 Department of Medicine, University of California, San Francisco, San Francisco, CA, USA; 6 Faculty of Medicine, Makerere University, Kampala, Uganda; 7 Department of Microbiology, Makerere University, Kampala, Uganda; 8 HIV/AIDS Division, University of California, San Francisco, CA, USA

## Abstract

**Background:**

Sputum concentration increases the sensitivity of smear microscopy for the diagnosis of tuberculosis (TB), but few studies have investigated this method in human immunodeficiency virus (HIV)-infected individuals.

**Methods:**

We performed a prospective, blinded evaluation of direct and concentrated Ziehl-Neelsen smear microscopy on a single early-morning sputum sample in HIV-infected patients with > 2 weeks of cough hospitalized in Kampala, Uganda. Direct and concentrated smear results were compared with results of Lowenstein-Jensen culture.

**Results:**

Of 279 participants, 170 (61%) had culture-confirmed TB. The sensitivity of direct and concentrated smear microscopy was not significantly different (51% vs. 52%, difference 1%, 95% confidence interval (CI): [-7%, 10%], p = 0.88). However, when results of both direct and concentrated smears were considered together, sensitivity was significantly increased compared with either method alone (64%, 95% CI: [56%, 72%], p < 0.01 for both comparisons) and was similar to that of direct smear results from consecutive (spot and early-morning) specimens (64% vs. 63%, difference 1%, 95% CI: [-6%, 8%], p = 0.85). Among 109 patients with negative cultures, one had a positive direct smear and 12 had positive concentrated smears (specificity 99% vs. 89%, difference 10%, 95% CI: [2%, 18%], p = 0.003). Of these 13 patients, 5 (38%) had improved on TB therapy after two months.

**Conclusion:**

Sputum concentration did not increase the sensitivity of light microscopy for TB diagnosis in this HIV-infected population. Given the resource requirements for sputum concentration, additional studies using maximal blinding, high-quality direct microscopy, and a rigorous gold standard should be conducted before universally recommending this technique.

## Background

Direct sputum smear microscopy is the cornerstone of tuberculosis (TB) diagnosis worldwide [1]. Direct smear microscopy is rapid, inexpensive [2-4], highly specific [5-7], and capable of identifying the most infectious cases of TB [7-9], but its sensitivity is limited, particularly in those with human immunodeficiency virus (HIV) co-infection [10-13]. Processing of sputum with subsequent concentration by centrifugation or sedimentation may increase the sensitivity of smear microscopy [14], and some investigators have recommended sputum processing and concentration as a global standard [15]. However, others have called for more evidence before implementing such a policy change, citing concerns such as increased cost for materials and training, higher biosafety requirements, and difficulty standardizing techniques across sites [16]. Of equally great concern, sputum concentration carries a risk of decreased sensitivity (e.g., through destruction of bacilli during concentration) [17,18] and specificity (e.g., through contamination during additional transfer steps) [19,20]. In addition, the difficulty in blinding readers as to whether a specimen is concentrated presents an inherent risk of bias in studies of sputum concentration. Few studies have employed the clear design and reporting requirements required to minimize this inherent risk [21] or evaluated sputum concentration in populations with high HIV prevalence [22-26].

We therefore performed a prospective, blinded evaluation of direct and concentrated smear microscopy – performed simultaneously on a single early-morning sputum specimen – in a population of hospitalized, HIV-infected patients with cough for 2 or more weeks in Kampala, Uganda.

## Methods

### Study Population

Consecutive HIV-infected patients admitted to the medical wards of Mulago Hospital (Kampala, Uganda) between September 2007 and April 2008 for respiratory illness with cough of at least 2 weeks' duration were eligible for the study. We included patients who provided informed consent and an early-morning sputum specimen for TB diagnosis. We excluded patients who were receiving anti-TB treatment or had clinical evidence of congestive heart failure. The study protocol was approved by the institutional review boards at Makerere University, Mulago Hospital, the Uganda National Council for Sciences and Technology, and the University of California, San Francisco.

### Patient Evaluation

All patients were tested for HIV infection with a sequential testing algorithm incorporating three rapid enzyme immunoassay kits. For TB diagnosis, patients provided a randomly-timed sputum sample for direct smear microscopy at the time of enrollment. In addition, patients provided an early-morning sputum sample on the morning following admission; this sample was sent for both direct and concentrated smear microscopy (as described below). Patients without any positive smear examinations were offered bronchoscopy with bronchoalveolar lavage (BAL) if the procedure was deemed safe and appropriate by the chest medicine consultant. All sputum and BAL specimens were sent for mycobacterial culture. Patients with suspected TB (determined by the treating ward physician) began treatment with isoniazid, rifampin, ethambutol, and pyrazinamide. Patients were evaluated during an outpatient visit or by telephone interview between two and four months after hospital discharge to assess for clinical/radiographic improvement.

### Laboratory Methods

Sputum and BAL samples were analyzed at the Uganda National Tuberculosis and Leprosy Programme Reference Laboratory (NTRL). Both direct and concentrated smears were prepared from the same specimen. Direct smears were prepared and stained using the hot Ziehl-Neelsen method (1% carbol-fuchsin dye) [27]. Specimens were then decontaminated with a 1% N-acetyl-L-cysteine (NALC)-2% sodium hydroxide (NaOH)-2% sodium citrate solution and concentrated by centrifugation at 3000 × g for 10 minutes [28]. The concentrated specimen was then used to prepare mycobacterial cultures by inoculation onto two separate Lowenstein-Jensen slants and, for the early-morning specimen only, a concentrated smear stained using the hot Ziehl-Neelsen method [29]. Concentrated smears were labeled with random identification numbers immediately after preparation so that readers could not determine if direct and concentrated smears were from the same patient.

NTRL staff, who were also blinded to all clinical information, read all smears within 48 hours of preparation using a standard light microscope (magnification 1000×). They reported the presence or absence of acid-fast bacilli (AFB) using the WHO/IUATLD scale, with a positive result corresponding to ≥ 1 AFB per 100 high-power fields (HPFs) [29]. They also read cultures weekly, designating as negative any slant with no growth after eight weeks of incubation. Positive cultures (defined as ≥ 1 colony forming units) were confirmed with Ziehl-Neelsen staining. Study staff promptly communicated all laboratory results to ward physicians responsible for treatment decisions. Since 2005, the Uganda NTRL has participated in a semi-annual external quality assurance program for smear microscopy administered by the World Health Organization and has passed all quality assurance assessments.

### Outcome Definitions

The primary outcome for our analyses was culture-positive TB, defined as a positive Lowenstein-Jensen (LJ) culture result from the randomly-timed sputum specimen, early morning sputum specimen, or, when available, BAL specimen. We performed two secondary analyses using different "gold standard" definitions of TB. First, we restricted the definition of TB to include only patients with a positive culture on the same specimen from which smears were prepared. Second, we broadened the definition of TB to also include patients who improved clinically on empiric TB therapy, as documented by a study medical officer and a chest consultant (W.W. or S.Y.) between two and four months after hospital discharge.

### Sample Size

We aimed to collect concentrated sputum specimens from 329 patients, in order to provide 90% power to detect a difference between 50% and 60% sensitivity for direct and concentrated sputum smear, respectively, assuming a 2-sided alpha of 0.05, phi (correlation coefficient) of 0.5, and a projected 20% dropout rate due to contamination or failure to perform culture (i.e., final sample size of 262 patients). Sample size calculations were performed using PS: Power and Sample Size Calculation, version 2.1.31 [30].

### Statistical Analysis

Analyses were performed using STATA 9.0 (Stata Corp., College Station, TX). Sensitivity and specificity were calculated in reference to the outcomes defined above, and compared between diagnostic strategies using McNemar's test. Bivariate comparisons were made using Fisher's exact test for dichotomous variables and the Wilcoxon rank-sum test for continuous variables. Concordance was measured using the kappa statistic. All p-values were two-sided, with statistical significance defined as p < 0.05.

## Results

### Study Population

**Figure 1:**
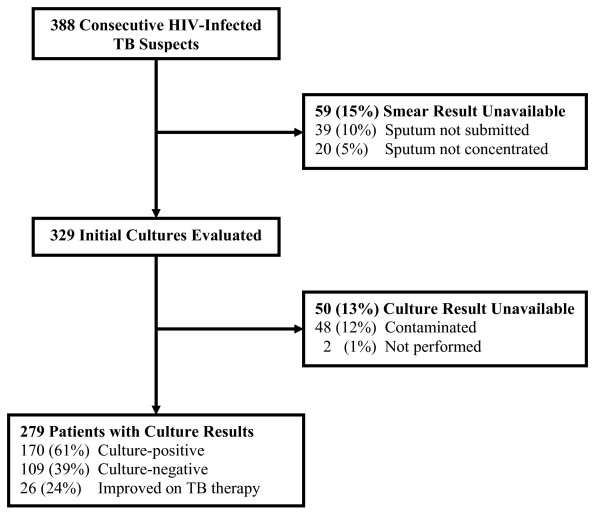
**Study Flow Diagram**.

Of 388 eligible patients, 39 (10%) were unable to provide an early-morning sputum specimen (unable or unwilling to spontaneously expectorate), 20 (5%) did not have a concentrated smear performed, 48 (12%) had a contaminated sputum culture, and 2 (1%) did not have culture performed despite the availability of concentrated smear, giving a final sample size of 279 HIV-infected TB suspects (Figure 1). The majority of exclusions (other than for contamination) occurred during a two-week period when the NTRL lacked sufficient staffing to process research samples in addition to their routine clinical work. Patients excluded from the study did not differ from those included with regard to baseline characteristics (gender, age, CD4 count, antiretroviral use) or the proportion of TB cases confirmed by positive direct or concentrated smear results (p > 0.15 for all comparisons). However, excluded patients were more likely to die in the hospital (28% vs. 8%, p < 0.001).

Of the 279 patients included in the study, 143 (51%) were positive for TB on culture of the early-morning sputum specimen used for comparison of direct and concentrated smear results. An additional 27 (10%) were positive on culture of another specimen. Of the remaining 109 patients, 103 (98%) had two or more negative cultures and six had only a single negative culture. Exclusion of these latter six patients did not materially affect results (all had negative direct and concentrated smears). Patients with at least one positive TB culture had significantly lower CD4+ T-lymphocyte counts than patients with negative TB cultures (median 33 vs. 101, p < 0.0001), but these two groups did not differ significantly by gender, age, education, antiretroviral use, or by mortality at hospital discharge or at two months (Table 1). Regarding sputum quality, of the 279 specimens, 33 (12%) were described as salivary, 191 (68%) mucoid, 41 (15%) purulent, and 14 (5%) bloody. Exclusion of salivary specimens or restriction to mucoid specimens reduced the sample size but did not materially affect results.

**Table 1:** Characteristics of Study Population, by Tuberculosis Culture Status

Characteristic	Total (n = 279)	Culture-positive (n = 170)	Culture-negative (n = 109)	p-value
Female gender, n (%)	166 (60)	98 (58)	68 (62)	0.46
Age, median (IQR)	34 (27–40)	33 (27–40)	34 (28–40)	0.88
Education beyond primary level, n (%)	97 (35)	61 (36)	36 (33)	0.70
CD4 count, median (IQR) (n = 274)	49 (15–169)	33 (12–115)	101 (31–219)	< 0.0001
Antiretroviral use on admission, n (%)	42 (15)	24 (14)	18 (17)	0.37
Mortality, n (%)				
In hospital (n = 279)	23 (8)	13 (8)	10 (9)	0.66
Two-month (n = 241)	65 (27)	43 (30)	22 (23)	0.30
Clinical presentation, n (%)				
Fever/chills/sweats	262 (94)	158 (93)	104 (95)	0.45
Weight loss	265 (95)	167 (98)	98 (90)	0.003
Hemoptysis	68 (28)	31 (21)	37 (40)	0.001

IQR, interquartile range

### Sensitivity and Specificity of Direct and Concentrated Sputum Smear Microscopy

As shown in Table 2, the sensitivity of direct and concentrated smear microscopy was similar when using any positive culture result as the gold standard (51% vs. 52%, difference 1%, 95% confidence interval: [-7%, 10%], p = 0.88). However, concordance between the two methods was only fair, both among all specimens (80% overall agreement; unweighted kappa 0.56, 95% CI: [0.44, 0.68]) and among culture-positive specimens (75% overall agreement; unweighted kappa 0.51, 95% CI: [0.35, 0.66]). As a result, the performance of both smear techniques on the same specimen yielded an absolute increase of 12–13% in sensitivity over either smear alone (p < 0.01 for increase in sensitivity over direct or concentrated smear alone). In addition, combining the results of direct and concentrated smears of a single specimen had a similar sensitivity to performing direct smear microscopy on two consecutive (randomly-timed and early-morning) sputum specimens (64% vs. 63%, difference 1%, 95% confidence interval: [-6%, 8%], p = 0.85). Among the 109 patients with negative cultures, one had a positive direct smear (specificity 99%, 95% CI: [94%, 100%]), and 12 had positive concentrated smears (specificity 89%, 95% CI: [81%, 95%], p = 0.003 for difference).

**Table 2:** Sensitivity and Specificity of Concentrated and Direct Smear, By Reference Outcome

Concentrated Smear Result	Direct Smear Result		Total
**Gold Standard: Sputum Culture**			

A. Sensitivity			

	Positive	Negative	
Positive	67 (39%)	22 (13%)	89 (52%)
Negative	20 (12%)	61 (36%)	81 (48%)
Total	87 (51%)	83 (49%)	170 (100%)
Sensitivity of Direct Smear = 0.51 (0.43–0.59)			
Sensitivity of Concentrated Smear = 0.52 (0.44–0.61)			
Sensitivity of Direct + Concentrated Smear = 0.64 (0.56–0.72)^*a*, *b*^			

B. Specificity			

	Positive	Negative	
Positive	0 (0%)	12 (11%)	12 (11%)
Negative	1 (1%)	96 (88%)	97 (89%)
Total	1 (1%)	108 (99%)	109 (100%)
Specificity of Direct Smear = 0.99 (0.94–1.0)^*a*^			
Specificity of Concentrated Smear = 0.89 (0.81–0.95)^*b*^			
Specificity of Direct + Concentrated Smear = 0.88 (0.80–0.94)^*b*^			

**Gold Standard: Sputum Culture + Clinical Criteria**			

A. Sensitivity			

	Positive	Negative	
Positive	67 (34%)	27 (14%)	94 (48%)
Negative	20 (10%)	82 (42%)	102 (52%)
Total	87 (44%)	109 (56%)	196 (100%)
Sensitivity of Direct Smear = 0.44 (0.36–0.52)			
Sensitivity of Concentrated Smear = 0.48 (0.40–0.56)			
Sensitivity of Direct + Concentrated Smear = 0.58 (0.50–0.66)^*a*, *b*^			

B. Specificity			

	Positive	Negative	
Positive	0 (0%)	7 (8%)	7 (8%)
Negative	1 (1%)	75 (90%)	76 (92%)
Total	1 (1%)	82 (99%)	83 (100%)
Specificity of Direct Smear = 0.99 (0.93–1.0)			
Specificity of Concentrated Smear = 0.92 (0.83–0.97)			
Specificity of Direct + Concentrated Smear = 0.90 (0.81–0.96)^*b*^			

^*a *^p < 0.05 for comparison with concentrated smear alone

^*b *^p < 0.05 for comparison with direct smear alone

We performed two secondary analyses using different definitions for the gold standard. First, when we restricted the gold standard definition to a positive culture result of the same specimen from which smears were prepared, the sensitivities of direct and concentrated smear remained similar (59% vs. 58%, p = 1.0), and the specificity of direct smear remained higher than that of concentrated smear (97% vs. 87%, p = 0.003). Second, when we expanded the gold standard to include patients with negative cultures but clinical response to TB therapy, the sensitivity of the two methods remained similar (44% vs. 48%, p = 0.38). However, the difference in specificity was no longer statistically significant (92% vs. 99%, p = 0.07) because 5 (42%) of the 12 patients with positive concentrated smears but negative cultures had documented clinical improvement on TB therapy at 2-month follow-up (Table 3).

**Table 3:** Data on Patients with Positive Smears and Negative Cultures

Patient ID	Admission CD4 Count	**Smear Result**^a^		Final Diagnosis	Treated for TB After Discharge?	Status 2 Months After Discharge
					
		Direct	Concentrated			
1	31		1+	TB	Yes	Improved
2	193		1+	TB	Yes	Improved
3	196		1+	TB	Yes	Improved
4	398		Scanty	TB	Yes	Improved
5	687		3+	TB	Yes	Improved
6	110		1+	Aspergillus	Yes	No Improvement
7	3		1+	PCP	Died in hospital	Dead
8	5		Scanty	Bacterial Pneumonia	No	Improved
9^*b*^	12	Scanty		Unknown	Died in hospital	Dead
10	31		1+	Unknown	Died in hospital	Dead
11	59		1+	Unknown	Died in hospital	Dead
12	81		Scanty	Bacterial Pneumonia	No	Improved
13	399		1+	Unknown	No	No Improvement

TB, tuberculosis; PCP, *Pneumocystis jirovecii *pneumonia

^*a *^Scanty = 1–9 acid-fast bacilli (AFB) per 100 high-power fields (HPF), 1+ = 1–9 AFB per 10 HPF, 3+ = >10 AFB per HPF.

^*b *^Direct smear alone was positive; for other patients, the concentrated smear alone was positive.

### Density of Acid-Fast Bacilli

Table 4 compares the density of AFB on specimens from patients with culture-confirmed TB. Of 170 confirmed TB patients, 67 (39%) were positive on both direct and concentrated smear, 61 (36%) were negative on both, 20 (12%) were positive only on concentrated smear, and 22 (13%) were positive only on direct smear. Among 20 specimens that were positive only after concentration, 8 (40%) had 10 or more AFB per 100 HPF. By contrast, among 22 specimens that were positive only on direct smear, 19 (86%) had 10 or more AFB per 100 HPF.

**Table 4:** Density of Acid-Fast Bacilli (per 100 High-Power Fields) in Patients with Culture-Positive Tuberculosis

		**Direct Smear Result**			**Total**
			
**Concentrated Smear Result**		0	1–9	≥ 10	
	0	61	3	19	**83**
	1–9	12	2	2	**16**
	≥ 10	8	7	56	**71**
**Total**		**81**	**12**	**77**	**170**

## Discussion

Sputum concentration did not increase the sensitivity of light microscopy for TB diagnosis in this prospective, blinded evaluation of 279 hospitalized, HIV-infected adults in Kampala, Uganda. Moreover, sputum concentration decreased specificity, though this difference was attenuated when clinical response to TB therapy was considered in the gold standard. The performance of concentrated smear microscopy requires further evaluation before recommending universal implementation of this technique.

Our finding that sputum concentration prior to Ziehl-Neelsen staining did not significantly increase sensitivity for TB over direct smear microscopy differs from the results of five prior studies that found higher sensitivity in HIV-endemic populations after sputum concentration [31-35]. This discrepancy may reflect differences in study site (e.g., research versus field setting), patient population (e.g., specially-selected outpatients versus consecutive hospitalized patients), gold standard (e.g., multiple cultures and follow-up data not routinely used versus used), or study methodology (e.g., sodium hypochlorite and sedimentation versus NALC-NaOH and centrifugation). In addition, although many studies employed some form of blinding, it is difficult to blind readers to processing method (direct vs. concentrated) due to the different appearance of unprocessed versus processed slides. This imperfect blinding is known to influence the results of sputum smear microscopy [36] and would be expected to inflate measured sensitivity for concentrated smear. Although we cannot exclude random chance or weaknesses in our study design as the explanation for our negative results, a number of additional considerations speak to the veracity of our findings. First, our reported 52% sensitivity of concentrated smear is similar to that in previous studies of HIV-infected individuals (i.e., 50% [37], 52% [38], and 54% [39]), suggesting that the discrepancy in findings may reflect higher sensitivity of direct microscopy in the present study, rather than higher sensitivity of concentrated microscopy in other studies. In addition, our study incorporated several features that strengthen its internal validity – consecutive enrollment of a relevant population, clear description of included and excluded patients, blinded reading of index and reference tests by trained non-research staff, and a gold standard which incorporated multiple culture results and follow-up assessment [40,41]. Thus, while our findings differ from those of previous studies, the present study provides sound evidence that sputum concentration does not universally increase smear sensitivity among HIV-infected TB suspects, and it suggests that provision of high-quality direct microscopy may diminish the benefit of sputum concentration.

In this study, direct and concentrated sputum smear had surprisingly low concordance among patients with culture-confirmed TB (kappa = 0.51), even though both techniques were performed on the same sputum specimen. Although direct and concentrated smear microscopy had similar sensitivities, they detected different patients: 39% (42/109) of all smear-positive patients with culture-confirmed TB were only positive by a single method (Table 2). Although most prior studies [42,43] have found moderate-to-high concordance between direct and concentrated smear, one earlier study [44] – also performed using high-speed centrifugation in an African reference lab – found results similar to those presented here (sensitivity of 43% for direct smear, 44% for concentrated smear, and 55% for direct plus concentrated smear). Collection of a single sputum specimen would reduce patient burden in terms of number of visits to health care centers, and could also decrease the delay between clinical presentation and initiation of treatment. Given the similar sensitivity of direct and concentrated smear microscopy also observed in our study, future studies should investigate whether performing multiple direct smears on a single specimen can substantially increase sensitivity for TB, avoiding the need for multiple patient visits.

As with any evaluation of smear microscopy, this study has certain limitations. First, our study was designed to measure a clinically relevant difference between direct and concentrated microscopy and was not powered to demonstrate equivalence. However, our 95% confidence intervals exclude more than a 10% absolute difference in sensitivity between the two methods. Second, this study was conducted in a national reference laboratory using NALC-NaOH on early-morning specimens collected from a population of HIV-infected, hospitalized patients. Thus, our results may not fully generalize to other settings (e.g., peripheral laboratories, laboratories using alternative sputum processing methods, non-HIV populations, and healthier outpatient populations). Estimates of diagnostic performance are known to vary between ambulatory and hospital settings. However, the choice of study population is less likely to impact a comparison between two diagnostic techniques. In addition, given the rigorous training required of microscopists at the Uganda NTRL, it is unlikely that laboratory inexperience explains the results of the present study. Finally, in order to better replicate actual test conditions, internal quality assurance was not performed during the study period. Though we would not expect reliability to differentially affect direct versus concentrated sputum smear results, we were unable to quantify inter-reader and intra-reader agreement.

## Conclusion

In conclusion, we failed to find a difference in sensitivity between direct and concentrated sputum smear microscopy performed in a national reference laboratory serving an HIV-infected hospitalized adult population. Before widely recommending sputum concentration, additional field evaluations that demonstrate benefit when incorporating strict blinding, high quality direct smear microscopy, and a clear gold standard are needed. Such studies should also investigate whether simpler modifications (e.g., parallel performance of multiple direct smears on a single sputum specimen) can similarly increase sensitivity and cost-effectiveness. Ultimately, modifications in smear microscopy may increase the yield of TB diagnosis only marginally, a possibility which emphasizes the need for development and testing of novel rapid diagnostic technologies.

## Abbreviations

TB: tuberculosis; HIV: human immunodeficiency virus; CI: confidence interval; BAL: bronchoalveolar lavage; NTRL: National Tuberculosis and Leprosy Programme Reference Laboratory; NALC: N-acetyl-L-cysteine; NaOH: sodium hydroxide; AFB: acid-fast bacilli; WHO: World Health Organization; IUATLD: International Union Against Tuberculosis and Lung Disease; HPF: high-powered field.

## Competing interests

The authors declare that they have no competing interests.

## Authors' contributions

AC and JLD participated in study design, data collection, statistical analysis, and drafting of the manuscript. DWD performed the primary statistical analysis and drafted the initial manuscript. WW, SY, MJ, and JM participated in study design, data collection, and drafting of the manuscript. PCH and LH participated in study design and drafting of the manuscript. All authors approved the final manuscript.

## Pre-publication history

The pre-publication history for this paper can be accessed here:

http://www.biomedcentral.com/1471-2334/9/53/prepub
